# Genome analysis and genomic comparison of a fungal cultivar of the nonsocial weevil *Euops chinensis* reveals its plant decomposition and protective roles in fungus-farming mutualism

**DOI:** 10.3389/fmicb.2023.1048910

**Published:** 2023-02-16

**Authors:** Wenfeng Guo, Wei Wang, Jun Tang, Tianyu Li, Xiaoqiong Li

**Affiliations:** ^1^Guangxi Key Laboratory of Forest Ecology and Conservation, College of Forestry, Guangxi University, Nanning, Guangxi, China; ^2^Guangxi Crop Genetic Improvement and Biotechnology Lab, Guangxi Academy of Agricultural Sciences, Nanning, Guangxi, China; ^3^Wuhan Benagen Technology Company Limited, Wuhan, Hubei, China

**Keywords:** *Euops*, nonsocial insect, fungus farming, mutualism, molecular, genome, *Penicillium*

## Abstract

Fungus-farming mutualisms are models for studying co-evolutionary among species. Compared to well-documented fungus-farming in social insects, the molecular aspects of fungus-farming mutualisms in nonsocial insects have been poorly explored. *Euops chinensis* is a solitary leaf-rolling weevil feeding on Japanese knotweed (*Fallopia japonica*). This pest has evolved a special proto-farming bipartite mutualism with the fungus *Penicillium herquei*, which provide nutrition and defensive protection for the *E. chinensis* larvae. Here, the genome of *P. herquei* was sequenced, and the structure and specific gene categories in the *P. herquei* genome were then comprehensively compared with the other two well-studied *Penicillium* species (*P. decumbens* and *P. chrysogenum*). The assembled *P. herquei* genome had a 40.25  Mb genome size with 46.7% GC content. A diverse set of genes associating with carbohydrate-active enzymes, cellulose and hemicellulose degradation, transporter, and terpenoid biosynthesis were detected in the *P. herquei* genome. Comparative genomics demonstrate that the three *Penicillium* species show similar metabolic and enzymatic potential, however, *P. herquei* has more genes associated with plant biomass degradation and defense but less genes associating with virulence pathogenicity. Our results provide molecular evidence for plant substrate breakdown and protective roles of *P. herquei* in *E. chinensis* mutualistic system. Large metabolic potential shared by *Penicillium* species at the genus level may explain why some *Penicillium* species are recruited by the *Euops* weevils as crop fungi.

## Introduction

A number of insects have established various associations with microorganisms, and among these, insect-fungus mutualisms are one of the better-studied in nature (Biedermann and Vega, 2020). Many eusocial and subsocial insects such as ants (Mueller et al., 2001), termites (Aanen, 2006), and ambrosia beetles (Kirkendall et al., 2015) exhibit sophisticated forms of mutualistic relationships with fungi (fungus-farming mutualisms) that allow these insects to cultivate their fungal crops on appropriate substrates, and in turn these insects obligatorily rely on consumption of their fungal crops for key nutrients and/or certain services such as protection from enemies (Biedermann and Vega, 2020). Mutualisms between fungi and fungus-farming insects are model systems for studying co-evolutionary interactions between species (Nygaard et al., 2016; Solomon et al., 2019; Biedermann and Vega, 2020; Pereira and Kjellberg, 2021). Compared to the well-documented fungus-farming mutualisms in some social insects, fungus farming by non-social organisms is uncommon, but includes some examples such as a lizard beetle *Doubledaya bucculenta* (Toki et al., 2012), weevils in the genus *Euops* (Coleoptera: Attelabidae) (Sawada and Morimoto, 1986; Kobayashi et al., 2008; Li et al., 2012), a marine snail (Silliman and Newell, 2003), and several species of damselfish (Hata and Kato, 2006). The biological and molecular aspects of fungus-farming mutualisms in solitary, non-social insects have been poorly explored.

Bioinformatic analysis of the genomic sequence of a fungal symbiote can be used to assist the discovery of molecular mechanisms responsible for symbiotic association, nutrition, pathogenicity, and/or defensive protection (Fan et al., 2015; Fu et al., 2020). Moreover, comparative genomics is a useful tool to better understand the molecular basis of fungus-farming mutualism in insects (Nygaard et al., 2011; Poulsen et al., 2014). Many molecular and genomic studies have provided new insights to our understanding of the establishment (Mueller et al., 2018), persistence (Schmidt et al., 2022), recognition and specificity adaptations (da Costa et al., 2019; Skelton et al., 2019; Goes et al., 2020) of fungus-growing insects and their mutualistic fungal symbiotes in recent years. The available evidence has shown that mutualistic symbiotes are crucial for the survival of the host insects (Schmidt et al., 2022), the generation of phenotypic diversity (Gohli et al., 2017; Solomon et al., 2019), and in the origin of various niches (Six, 2020). Fungus-farming mutualisms can result in specialized organs with unique development (Mayers et al., 2022), novel metabolic capabilities (Huang et al., 2019; Schmidt et al., 2022), better defense against natural enemies or other stresses (Pathak et al., 2019), and contraction or expansion of novel genes (Nygaard et al., 2016). Elucidating how fungus-farming mutualisms influence genes and genomes is essential to better understand fungal evolution from a molecular sequence perspective.

Weevils of the genus *Euops* (Attelabidae) present interesting model systems for studying fungus-farming mutualism in nonsocial insects (Sakurai, 1985; Kobayashi et al., 2008; Grebennikov and Leschen, 2010; Li et al., 2012). All species of this genus have developed mutualistic relationships with fungi (Sawada and Morimoto, 1986). *Euops chinensis* Voss (Coleoptera: Attelabidae) is a solitary leaf-rolling weevil on Japanese knotweed *Fallopia japonica* (Houtt.) Ronse Decraene (Wang et al., 2010). This pest has evolved a special proto-farming bipartite mutualism with the fungus *Penicillium herquei* that the female adults carry the fungus in a specialized fungus transport organ called mycetangium (Francke-Grosmann, 1967; Grebennikov and Leschen, 2010), and inoculate the fungal spores to the leaf-rolls before laying eggs (Li et al., 2012). Interesting, the other two *Euops* leaf-rolling weevils, *Euops lespedezae* (Kobayashi et al., 2008) and *Euops splendida* (Sakurai, 1985), have also been reported to form fungus-farming mutualisms with *Penicillium* spp. However, *Euops* - *Penicillium* system remains to be poorly studied compared to other fungus-farming insects like ants and termites.

Although *E. chinensis* is obligatory dependency on *P. herquei* for nutrition and protection like other fungus-farming insects, its fungus proto-farming behavior pparently differs from that of social insects in that the female weevils do not continuously tend the cultivated material as the leaf-rolls are cut from the plants and gradually decay (Li et al., 2012; Wang et al., 2015), and therefore, *P. herquei* has a saprophytic life style when growing on the leaf-rolls. This fungal cultivar has been demonstrated to be vertically transmitted by the weevil (Li et al., 2016), and obligatorily benefit the development of *E. chinensis* by altering the chemical composition of leaf-rolls (Li et al., 2012) and protecting leaf-rolls against plant pathogens (Wang et al., 2015), it also acts as a food resource for the larvae (Wang et al., 2010; Li et al., 2012). Thus, the growth, development, or survival of *E. chinensis* are strongly influenced by *P. herquei* (Li et al., 2012).

In this study, we provide the first detailed description of the genome of *P. herquei*. The structure, metabolic capabilities, secondary metabolite gene clusters, and important pathogenic characteristics of the *P. herquei* genome were then comprehensively compared with the other two well-studied *Penicillium* species, *P. chrysogenum* and *P. decumbens*. The results would provide new insights into the molecular basis of fungus-farming mutualism in *Euops* - *Penicillium* system.

## Materials and methods

### Fungal isolation and culture conditions

Leaf-rolls constructed by *E. chinensis* were collected from Jiangxi Province (N27^°^46′16.33, E114^°^23′38.30), China, in early May in 2021. *Penicillium herquei* on the leaf-rolls was isolated according to the methods of Li et al. (2012), and the fungus was cultured on potato dextrose agar (PDA) plates. The strain *Penicillium herquei* XQL_2021 was purified by single-spore isolation, and was preserved at −80°C prior to use. For genome DNA extraction and sequencing, the fungal strain was cultured on PDA plates for 2 weeks at 25°C, the spores were then harvested by flooding with sterile distilled water, a 1-ml aliquot of a spore suspension was added to 20 ml potato dextrose broth (PDB) medium in 50-ml conical flasks and was cultured at 25°C on a rotary shaker at 200 rpm for 3 days. Fungal hyphae were collected in sterile tubes by filtering the culture liquid thoroughly with sterilized water. The hyphae collected were then washed thoroughly with sterilized water, immediately frozen with liquid nitrogen, and stored at −80°C until used.

### Genomic DNA extraction and sequencing

About 2.0 g aliquots of hyphae (fresh weight) was collected, and genomic DNA was extracted based on the cetyltrimethylammonium bromide (CTAB) methods (Porebski et al., 1997). The quantity and quality of the extracted genomic DNA were checked using a Nanodrop (Thermo Scientific, United States).

For short reads, sequencing was done on a NovaSeq 6000 platform, and a large fragment library was prepared using a NEBNext^®^ Ultra™ II DNA Library Prep Kit for Illumina (NEB, United States). The library was quantified using an Agilent 2100 bioanalyzer instrument (Agilent DNA 1000 reagents; Agilent, Santa Clara, CA, United States) and real-time quantitative PCR (RT-qPCR). The qualified libraries were amplified within the flow cell on an Illumina cBOT instrument for cluster generation (NovaSeq 6000 PE cluster kit; Illumina). The clustered flow cell was loaded onto a NovaSeq 6000 sequencer (NovaSeq 6000 SBS kit; Illumina) for paired-end sequencing with recommended read lengths of 150 bp. Raw reads were filtered using the SOAPnuke (v2.1.4) tool^1^ to remove reads with adaptors or unknown nucleotides, and low-quality reads with ≥50% low-quality bases. After data filtering, clean data were used for subsequent analyses.

For long reads, sequencing was done on a Nanopore PromethION platform, and the libraries were prepared with an Oxford Nanopore ligation kit (SQK-LSK109) according to a standard protocol. The purified library was loaded onto a primed R9.4 Spot-On Flow cell (FLO-MIN106), and sequencing was performed with a PromethION sequencer (Oxford Nanopore Technologies, Oxford, United Kingdom) running for 48 h at Wuhan Benagen Technology Company Limited (Wuhan, China). Resulting FAST5 files were base-called using the Oxford Nanopore GUPPY software (v0.3.0), and reads with a quality ≤7 were discarded.

### Genome assembly and annotation

Genomic assembly was performed using NECAT^2^. Two rounds of error correction were performed using Racon (v1.4.3) (Vaser et al., 2017) and Pilon (v1.23) based on the nanopore and the Illumina Novaseq sequencing data (Walker et al., 2014), respectively. The heterozygous sequences were removed using the Purge_haplotigs pipeline (v1.0.4) (Roach et al., 2018). Homology-based gene prediction was performed using SNAP (Johnson A.D. et al., 2008), AUGUSTUS v 3.2.1 (Stanke et al., 2006), and GeneMark-ES v4.21 (Ter-Hovhannisyan et al., 2008). BUSCO (v4.1.2) based on the fungi_odb10 reference database was employed to evaluate the quality of the prediction (Waterhouse et al., 2018). The tRNA regions and secondary structures were detected using tRNAscan-SE v1.23. The rRNAs were analyzed using RNAmmer software (Lagesen et al., 2007), and the small RNAs were predicted using Infernal v1.1.2 to search against the Rfam 9.1 database (Gardner et al., 2009). To evaluate the transposable elements within the *P. herquei* genome, the transposable elements were searched with the Repbase database (Bao et al., 2015) using RepeatMasker v4.0.9^3^.

For functional annotation, BLASTP searched against a series of protein databases, including UniProt/Swiss-Prot (Bairoch et al., 2005), Non-Redundant Protein Sequence Database (NR) in NCBI^4^, Gene Ontology (GO) (Gene Ontology Consortium, 2004), Kyoto Encyclopedia of Genes and Genomes (KEGG) (Kanehisa and Goto, 2000), and Cluster of Orthologous Groups (COG) (Tatusov et al., 2003) with a cut-off of <1e–05, and the best hit was used to infer the gene’s biological function.

### Gene orthology and phylogenetic analysis

The genome reference sequences of the other 14 Ascomycetes species (see Supplementary Tables S1, S2 in the supplemental materials) were downloaded from the NCBI database and were used to construct gene families. Sequence alignment was done with MUSCLE (Edgar, 2004), and positions containing gaps of ≥80% in multiple sequence alignment were trimmed using TrimaAI v1.4. rev22 (Capella-Gutiérrez et al., 2009). The comparison and annotation of orthologous gene clusters were carried out using OrthoFinder 2.2.7 (Emms and Kelly, 2019). Phylogenetic trees were constructed using the maximum-likelihood approach implemented in RAxML v8.0 (Stamatakis, 2014), the sequence of *Coccidioides immitis* (Stiles) (GenBank accession number GCA_000146045.2) was used as an outgroup. Gene family expansion and contraction were identified using CAFE v4.2 (De Bie et al., 2006). A time-calibrated phylogeny was inferred under the Bayesian framework employing fossil information (Drummond and Rambaut, 2007). The chronograms shown were calculated using the median clade credibility tree and 95% confidence intervals. The PAML mcmctree v4.5 program (Yang and Rannala, 2006) was used to compute split times using the approximate likelihood calculation algorithm. The model of sequence evolution was determined using Modeltest 3.7^5^. Tracer v1.5.0^6^ was applied to examine convergence, and two independent runs were performed for confirmation.

### Comparison of genomes of the three *Penicillium* species

For a better understanding of genomic characters of *P. herquei* within the context of the genus *Penicillium*, the genome of *P. herquei* was compared with the genomes of the other two well-studied *Penicillium* species, *P. chrysogenum* (GenBank accession no.: GCA_000149335.2) and *P. decumbens* (GenBank accession no.: GCA_002072245.1). Gene families were generated by MCL software (v12-068) using an inflation value of 2.0 (Enright et al., 2002). BLASTp was used to compare all protein sequences from the 3 selected species, the results were filtered using threshold limits of e values ≤1e–5, alignment identity ≥30%, and an alignment coverage ≥50%. GO annotation was performed by the use of Blast2GO, which assigned homologous sequences aligned by BLAST with Uniprot and the NR database to GO terms. The number of shared and specific gene families among the three *Penicillium* species were than analyzed.

### Specific gene categories annotation in the genomes of the three *Penicillium* species

Genes related to cellulose and hemicellulose degradation: Carbohydrate-active enzymes (CAZymes) in the three *Penicillium* species were annotated using BLAST (Johnson M. et al., 2008). The dbCAN annotation program HMMER 3 (Finn et al., 2011) was used to search against the CAZy (carbohydrate-active enzyme) database (Lombard et al., 2014). The results were combined when e values ≤1e–5. The class II peroxidases and dye-decolorizing peroxidases were further confirmed by BLAST searches against PeroxiBase (Fawal et al., 2012).

Secondary metabolism genes: Candidate transporter genes in the three *Penicillium* species were identified based on searches of the Transporter Classification Database (TCDB) (Saier et al., 2014) with e values ≤1e–5 and identity values ≥40%. The secondary metabolism biosynthesis genes and gene clusters in the genomes of the three *Penicillium* species were predicted with AntiSMASH 6.0 (Blin et al., 2021). The Comprehensive Antibiotic Research Database (CARD) (McArthur et al., 2013) was used to compare coding genes (of the three *Penicillium* species) involved in antimicrobial resistance.

Virulence associated genes: Candidate pathogen-host interactions (PHI) genes within the genome of the three *Penicillium* species were identified using BLASTp to search against PHI-base v4.3^7^, and protein alignments were performed to identify putative virulence-associated genes in the three *Penicillium* species with identity ≥40% and query coverage ≥70%.

## Results

### General genomic structure of *Penicillium herquei*

After quality control, we obtained 10.829 Gb of NovaSeq data (269 coverage) and 16.343 Gb of Nanopore data (406 coverage). Combined sequences from the two platforms were assembled into 65 scaffolds with an N_50_ value of 414,225 bp to obtain a total genome size of 40.25 Mb (46.72% GC content) (Table 1). We predicted 14,532 genes with an average length of 2,275 bp, and 96.12% of the protein-coding genes had significant sequence similarity to previously documented fungal sequences (Table 1). BUSCO was used to calculate the completeness of assembly and annotation. Among 1875 single-copy orthologs, 75.6% of contigs were complete and 16.4% of contigs were complete duplicated BUSCOs, while only 0.7% were fragmented and 7.4% were missing.

**Table 1:** Genome features of *Penicillium herquei*.

Assembling parameters	Values	Annotation parameters	Values
Total genome size (Mb)	40.25	# of genes	14,532
# of scaffolds	65	# of annotated genes	13,969
Maximum scaffold length (bp)	1,632,525	# of exon	49,056
Minimum scaffold length (bp)	131,442	# of intron	34,524
Depth of genome coverage	99.43%	# of ncRNAs	272
GC content (%)	46.7	# of tRNA	185

### Gene assembly and annotation

Of the predicted genes, 8,751 (60.22%) showed similarity to known proteins in the NR database, and 70.87% of these genes show significant matches to known proteins of 9 *Penicillium* species (Supplementary Table S3). We detected 49,056 exons with an average length of 421.53 bp. The average length of the introns was 100.23 nucleotides. For noncoding RNAs (ncRNAs), 185 tRNA, 41 RNA, and 40 snRNA genes were identified in the *P. herquei* genome (Supplementary Table S4). We identified 458,687 bp of repetitive elements in the *P. herquei* genome (1.14% of the genomic sequence). Tandem repeat sequences accounted for 0.04% and transposable elements for 1.69% of the assembled genome, and unknown and other repetitive elements accounted for 0.02% of the genome. Long terminal repeats (LTRs) were the most abundant transposable elements and accounted for about 0.43% of the genomic sequence (Supplementary Table S4).

**Figure 1:**
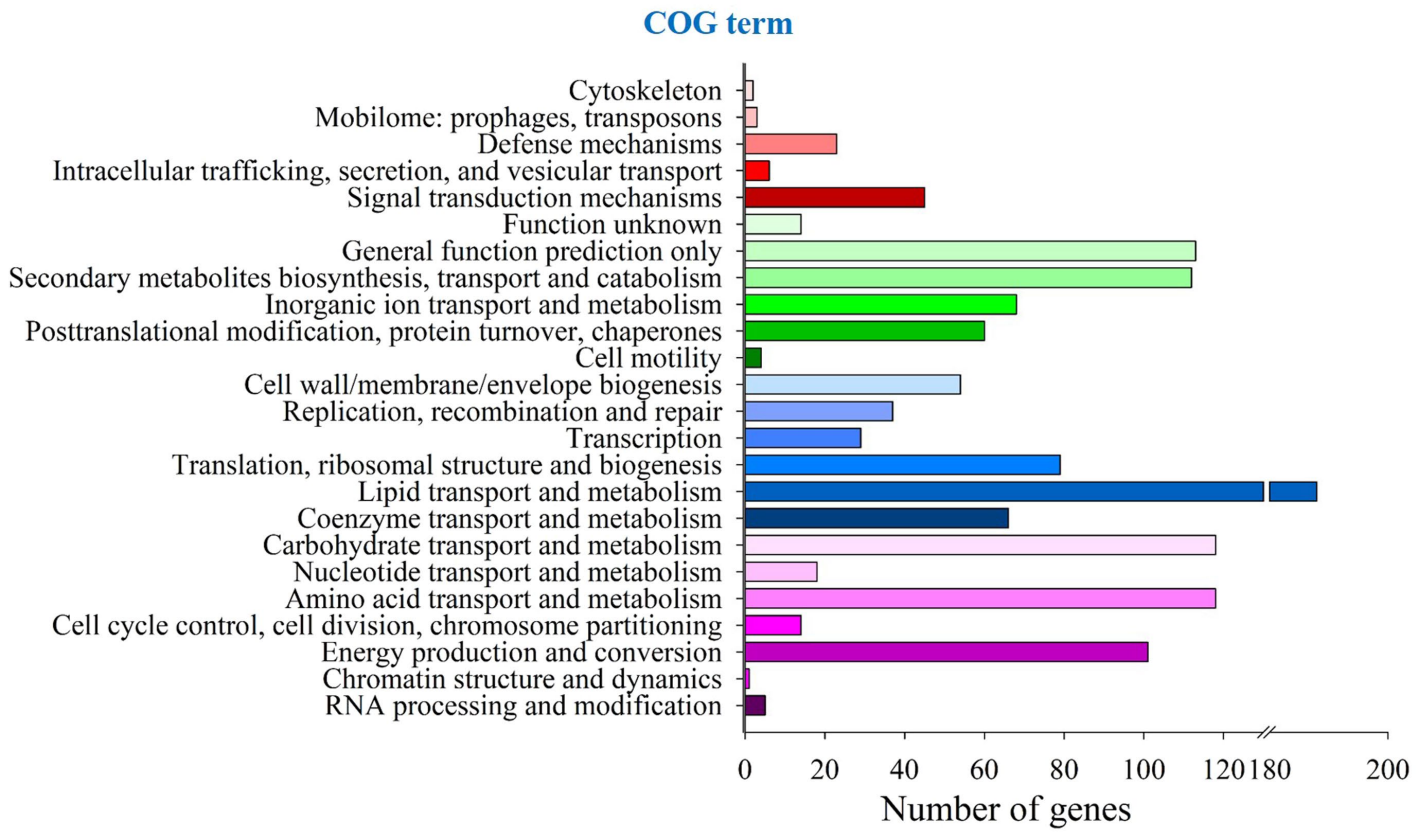
COG functional annotation of coding sequences in the whole genome of *Penicillium herquei*.

COG annotation results showed that 1,227 genes were classified into 24 gene types, accounting for 8.44% of the total genes in *P. herquei*, as shown in Figure 1. The functional annotation results in the GO database showed that 8,665 genes (59.63% of all the genes) could be classified into three types, with 7,114 being genes related to cellular components, 7,585 being genes related to molecular functions, and 7,086 being genes related to the biological processes. The GO terms with the highest numbers of genes that were classified as related to biological processes were genes related to transmembrane transport (405 genes). The GO terms with the highest numbers of genes that were classified as related to cellular components were the nucleus (2055 genes) or were integral components of membranes (641 genes). The GO terms with the highest numbers of genes classified as related to molecular function were for ATP binding (944 genes, 16.17%) and metal ion binding (873 genes, 14.95%) (Supplementary Figure S1). The results of KEGG pathway analysis showed that 6,638 (45.67%) genes were classified as related to 338 known metabolic pathways. The metabolic pathway with the largest number of genes was amino acid metabolism (771 genes), followed by carbohydrate metabolism (743 genes) and signal transduction (593 genes). Cluster analysis showed that the relevant 338 metabolic pathways could be categorized into the five groupings of metabolism, genetic information processing, organismal systems, cellular processes, and environmental information processing (Supplementary Figure S2).

### Gene orthology and phylogenetic analysis

**Figure 2:**
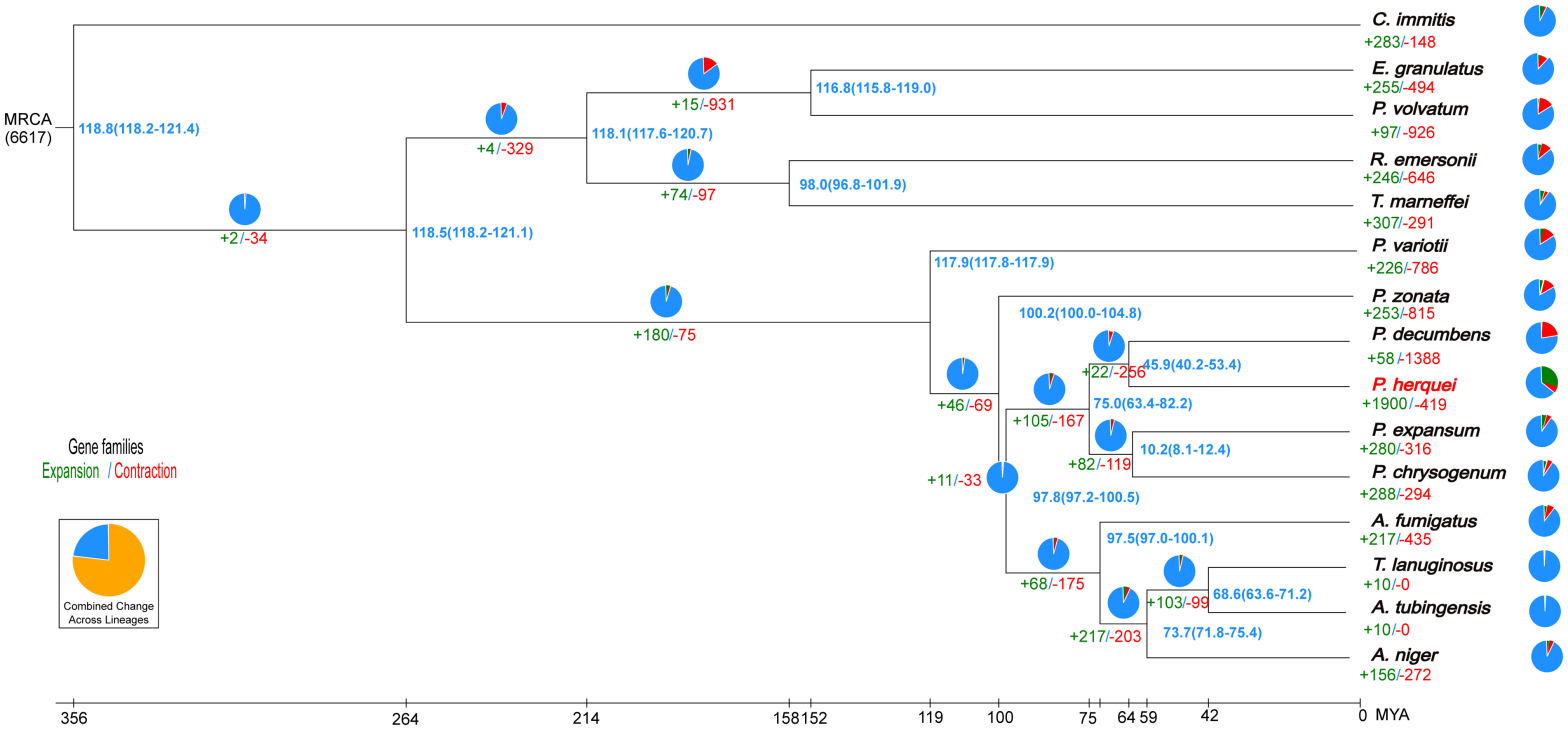
Phylogenetic tree of *Penicillium herquei* and sequences of the 14 fungal species available from GenBank. The blue numbers on the nodes are divergence times in units of million years ago. Numbers for significantly expanded (+, green) and contracted (−, red) gene families are shown below branches or taxon names with percentages indicated by pie charts.

OrthoFinder identified 2,852 gene clusters, among which were included 48,701 orthologous genes shared among all the 15 fungal species considered. From these shared gene clusters, 23,220 single-copy orthologous genes were chosen to analyze the evolutionary relationship of *P. herquei* with the other 14 fungal reference genomes. It is noteworthy that the *P. herquei* genome possesses more multiple-copy orthologs but fewer sigle-copy orthologs than the genomes of other 14 fungal species examined (Supplementary Figure S3). Phylogenetic analysis revealed that *P. herquei* clustered with other *Penicillium* species and was closest to the plant-pathogenic fungus *P. decumbens* (Figure 2). Moreover, 49 genes and 419 gene families were significantly contracted but 4,878 genes and 1900 gene families were significantly expanded in the *P. herquei* genome. Compared with other fungal species, *P. herquei* had the highest ratio of expanded gene families to contracted ones (Figure 2).

### Comparative genomics of three *Penicillium* species

**Figure 3:**
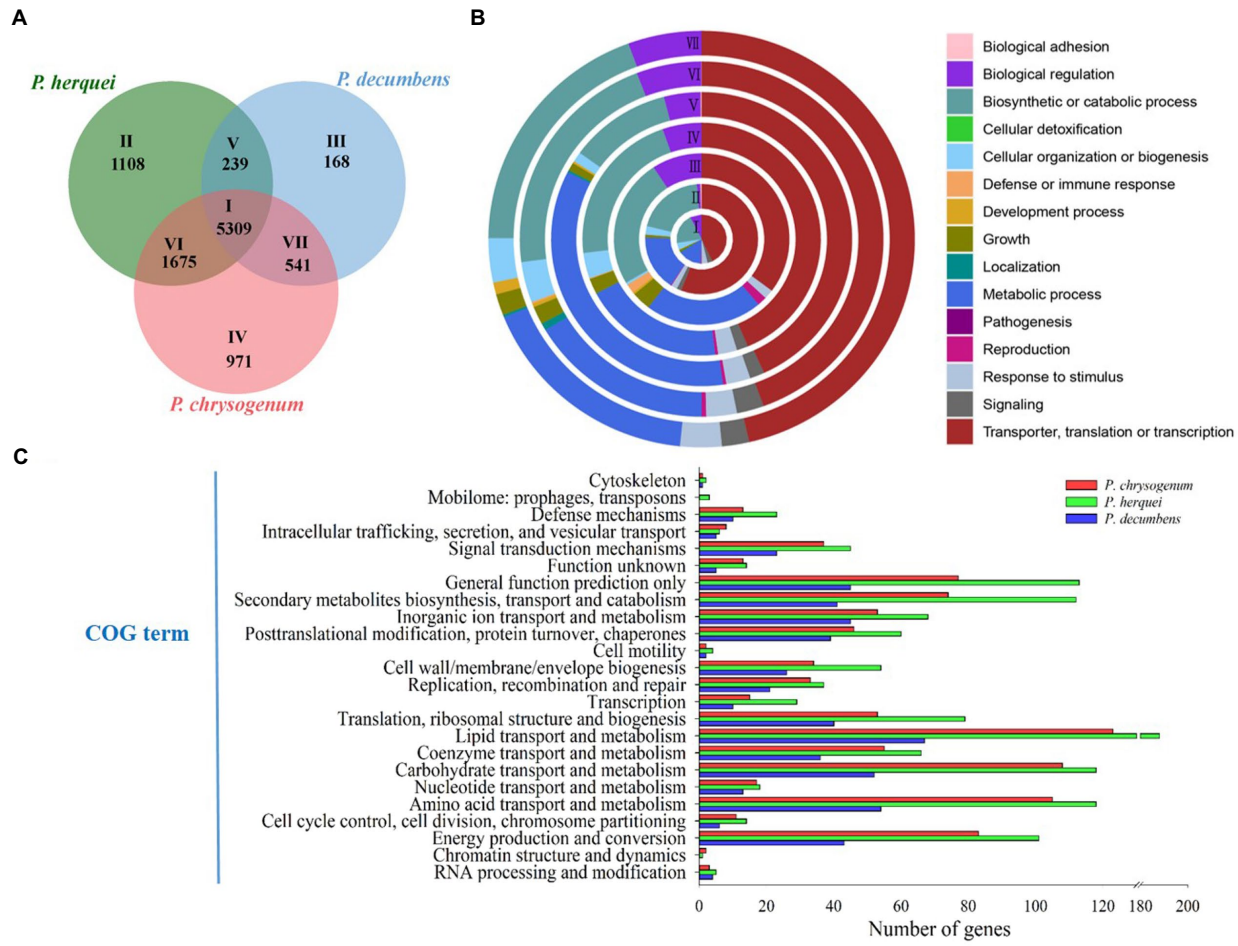
**(A)** Venn diagram showing the number of shared and specific gene families among three *Penicillium* species, *P. herquei* (*Pher*), *P. chrysogenum* (*Pchr*), and *P. decumbens* (*Pdec*). **(B)** Gene Ontology (GO) categories of gene families according to biological process. Circles I to VII correspond to areas I to VII in A. **(C)** Comparative analysis of genes by functional Cluster of Orthologous Groups (COG) categories, from the genomes of *P. herquei*, *P. chrysogenum*, and *P. decumbens*.

Genome size (40.25 Mb) was larger but GC content (46.7%) was lower for *P. herquei* compared to the other two *Penicillium* species examined (*P. chrysogenum*: 32.52 Mb with 48.9% GG content; *P. decumbens*: 23.94 Mb with 50.2% GC content). The three *Penicillium* species shared 5,309 gene families (Figure 3A). In addition to the core gene families that are all present in the three species, *P. herquei* shared more gene families with *P. chrysogenum* than with *P. decumbens*. There were 239 gene families that were uniquely shared *P. herquei* and *P. decumbens*, while 1,675 gene families were uniquely shared by *P. herquei* and *P. chrysogenum*. The shared and specific gene families in the three *Penicillium* species were classified by GO analysis according to their related biological processes (Figure 3B). The majority of the 5,309 core families (Circle I) were classified into the functional categories of cellular and metabolic processes, but approximately half of the species-specific gene families (Circles V to VII) could not be assigned into a designated GO category (Figure 3B). The results of COG annotation showed that *P. herquei* had more genes involved in matter transportation and metabolism, including those that clustered under the groups of “lipid transport and metabolism,” “carbohydrate transport and metabolism,” “amino acid transport and metabolism,” and “secondary metabolite biosynthesis, transport, and catabolism” (Figure 3C).

### Specific gene category annotations in the genomes of the three *Penicillium* species

**Figure 4:**
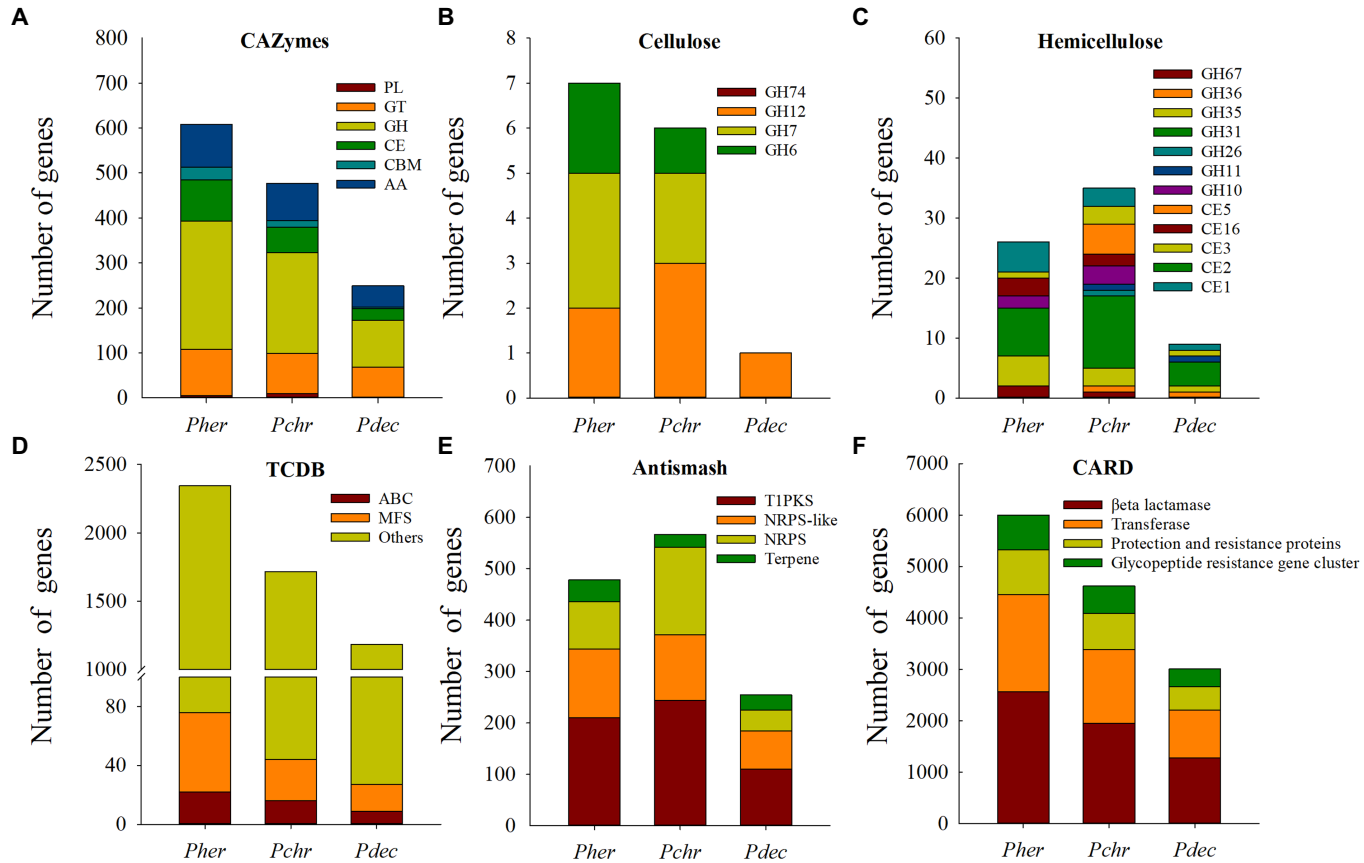
Comparison of genes encoding carbohydrate active enzymes (CAZymes) **(A–C)** and secondary metabolites **(D–F)** in *Penicillum herquei* (*Pher*), *Penicillium chrysogenum* (*Pchr*) and *Penicillium decumbens* (*Pdec*). **(A)** CAZymes identified in the genome of *Pher*, *Pchr* and *Pdec*. PL, polysaccharide lyase; GT, glycosyltransferase; GH, glycoside hydrolase; CE, carbohydrate esterase; CBM, carbohydrate-binding module; AA, auxiliary activity. CAZymes involved in cellulose degradation **(B)** and hemicellulose degradation **(C)**. **(D)** Secondary metabolite backbone genes. PKS, polyketide synthase; NRPS, nonribosomal peptide synthetase. **(E)** Genes encoding transporters. MFS: major facilitator superfamily of transporters, ATP-binding cassette (ABC) transporters. **(F)** Antimicrobial resistance genes identified in CARD database.

Genes related to cellulose and hemicellulose degradation: We identified 608 CAZymes in the *P. herquei* genome, consisting of 285 glycoside hydrolases (GHs), 103 glycosyl transferases (GTs), 95 enzymes with auxiliary activities (AAs), 92 carbohydrate esterases (CEs), 28 carbohydrate-binding modules (CBMs), and 5 polysaccharide lyases (PLs) (Figure 4A). *Penicillium herquei* had more CAZymes genes (especially for genes encoding GHs, CEs, and AAs) than the other two *Penicillium* species (*P. chrysogenum*, 477 and *P. decumbens*, 249) (Figure 4A). Moreover, many genes encoding for GHs and CEs related to cellulose and hemicellulose degradation were also found in the *P. herquei* genome. In particular, there were more GH6 and GH7 genes related to cellulose degradation as well as GH35, GH67, CE16 and CE1 genes related to hemicellulose degradation in the *P. herquei* genome than in the other two *Penicillium* species (Figures 4B,C).

Secondary metabolism genes: We identified 2,346 transport proteins in the *P. herquei* genome, more than in the other two *Penicillium* species. Among these transporters, 54 genes encoded major facilitator superfamily (MFS) proteins and 22 encoded ATP-binding cassette (ABC) proteins (Figure 4D). AntiSMASH analysis revealed that the number of secondary metabolite genes predicted for the *P. herquei* (520) genome was comparable with the number of these genes in the *P. chrysogenum* genome (566), but was higher than in the *P. decumbens* genome (254). These secondary metabolite genes included genes encoding type-I polyketide synthase (T1PKS), nonribosomal peptide synthetases (NRPS), and nonribosomal peptide synthetase-like (NRPS-like) enzyme (Figure 4E). In particular, the *P. herquei* genome contained 42 terpene synthase genes, which was more than the number of such enzymes in the other two *Penicillium* species (Figure 4E). CARD identified four antimicrobial resistance genes in the genomes of the three *Penicillium* species, including βeta lactamase, transferase, protection and resistance proteins, and a glycopeptide-resistance gene cluster. The numbers of these genes in *P. herquei* were all higher than in the other two *Penicillium* species (Figure 4F).

**Figure 5:**
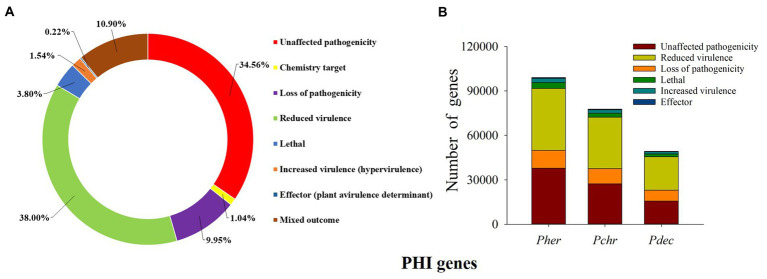
Putative pathogen-host interactions (PHI) genes. **(A)** The distribution of phenotypic categories of PHI gene orthologs in the *Penicillium herquei* genome. **(B)** Comparison of PHI genes in the three *Penicillium* species, *P. herquei* (*Pher*), *P. chrysogenum* (*Pchr*), and *P. decumbens* (*Pdec*).

Virulence associated genes: We predicted a total of 99,031 PHI genes in the *P. herquei* genome; the highest proportion of the PHI genes was related to “reduced virulence” (38.00%), followed by “unaffected pathogenicity” (34.65%), “loss of pathogenicity” (9.95%), and “mixed outcome” (10.90%) (Figure 5A). Although *P. herquei* had more PHI genes than *P. chrysogenum* (77,638) and *P. decumbens* (49,326), PHI genes related to reduced virulence, unaffected pathogenicity, or loss of pathogenicity were all more abundant in the *P. herquei* genome than in the other two *Penicillium* species (Figure 5B).

## Discussion

The assembly quality highly depends on the genome size. We found the genome size of *P. herquei* (40.25 Mb) was larger than that of the two *Penicillium* species examined, *P. chrysogenum* (23.94 Mb) and *P. decumbens* (32.52 Mb). Similarly, *Raffaelea ambrosiae*, a symbiote of *Platypus* ambrosia beetles, had a larger genome size (40.78 Mb) (Vanderpool et al., 2018) than that of its close non-ambrosia relative, *Ophiostoma piceae* (32.84 Mb) (Haridas et al., 2013). However, *Ambrosiella cleistominuta*, the fungal symbiote of the ambrosia beetle *Anisandrus maiche*, had a similar genome size (27.08 Mb) (Wilken et al., 2020) with its close non-ambrosia relative *Ceratocystis fimbriata* (31.61 Mb) (Santos et al., 2020). As many of fungi are highly heterozygous and polyploid, the assemblies of their genomes are often highly fragmented, and the different assemblers may generate different assembly sizes. Kooij and Pellicer (2020) considered that published fragmented genome assemblies have overestimated the genome sizes of polyploid fungal symbiotes.

The genome and gene evolution of fungi enable them to exist in diverse environments, patterns of gene family expansion or contraction can reflect particular selection pressure that a species has been subjected to over evolutionary time scales (Nygaard et al., 2011, 2016). Both gene family expansion and reduction were found in the *P. herquei* genome, consistent with the genomes of other fungal cultivars of termites (Poulsen et al., 2014) and leaf-cutting ants (Aylward et al., 2013). For example, the genome of the *Termitomyces* sp., cultivated by the termite *Macrotermes natalensis*, showed 10 gene family expansions but 4 gene family contractions (Poulsen et al., 2014). The fungus *Leucoagaricus gongylophorus*, cultivated by leaf-cutting ants has an expansion of lignocellulases (Aylward et al., 2013). The expansion or contraction of gene families has likely been driven by mutualisms between fungus-farming insects and their obligate cultivars (Poulsen et al., 2014; Nygaard et al., 2016). Evolutionary modifications in the attine ant genomes include unprecedented rates of genome-wide structural rearrangement, early loss of arginine biosynthesis and positive selection on chitinase pathways, correspondingly, reciprocal genomic evolution of their fungal cultivars includes loss of a key ligninase domain, changes in chitin synthesis and a reduction in carbohydrate-degrading enzymes (Nygaard et al., 2016).

*Penicillium herquei* belongs to *Penicillium* section *Sclerotiora*, although fungal species in this section are commonly found in soil, plants, and insects (Houbraken and Samson, 2011), phylogeny of this section is still poorly investigated. Moreover, the genus *Penicillium* is affiliated to the family Aspergillaceae, and it contains two subgenera, *Aspergilloides* and *Penicillium* (Houbraken and Samson, 2011). We have also noticed that *P. herquei* belongs phylogenetically to *Aspergilloides*, whereas the other two *Penicillium* species we compared belong phylogenetically to subgenus *Penicillium* (Houbraken and Samson, 2011). Thus, future study of genomes of other closer relatives in subgenus *Aspergilloides* would be helpful for learning more about *P. herquei*.

The plant cell wall is an important barrier against insect attack, however, most of insects lack endogenous enzymes for plant cell wall digestion (Tokuda, 2019). To overcome the barrier of the plant cell wall, many insects have established close associations with fungi (Calderón-Cortés et al., 2012), because of which can secrete a diverse array of enzymes, such as cellulose, hemicellulose, and pectin etc., that are capable of degrading cell wall polymers (Kubicek et al., 2014). Among these, CAZymes are responsible for the metabolism of glycoconjugates, oligosaccharides, and polysaccharides, and they play crucial roles in the synthesis and degradation of carbohydrates (Hage and Rosso, 2021) and in host-pathogen interactions (Kubicek et al., 2014). In our analysis, *P. herquei* had more CAZymes-related genes than did the two *Penicillium* species examined and it also has a large number of genes associated with cellulose and hemicellulose degradation, which play important roles in effectively degrading plant cell walls into nutrients such as glucose, mannose, galactose, acetic acid, and xylose (Knowles et al., 1987; Yang et al., 2007). These genes help degrade cell walls of the leaf-rolls that the larvae of *E. chinensis* consume, providing additional nutrients for the growth of the larvae. We have also demonstrated that *P. herquei* can alter leaf chemical components by lowering the cellulose content of the leaf-rolls (Li et al., 2012). GHs are common enzymes that can degrade cellulose, hemicellulose, and starch, and they are involved in the hydrolysis of the glycosidic bond between or within carbohydrate molecules (Wyman et al., 2005; Sammond et al., 2012). In our study, genes encoding GH7, GH6, GH35, and GH67 class enzymes outnumbered those encoding other GH enzymes, indicating that this fungus has a stronger ability to degrade cellulose and hemicellulose than the other two *Penicillium* species studied.

The higher number of CAZymes-related genes and genes associating with cellulose and hemicellulose degradation in the *P. herquei* genome may suggest it natural history. Besides of being a symbiote of *E. chinensis*, *P. herquei* occurs widely in soil, litter, fruits, and as plant endophytic fungus in nature (Tansakul et al., 2014; Zhou et al., 2019). Thus, this fungus has possibly evolved these genes associating with plant decomposition to thrive in natural environment, but keeps these genomic features after cultivated by *E. chinensis* as a fungal crop. Further work on comparison of both gene expression and compound production between *P. herquei* isolates from *E. chinensis* mycantagium and free-living counterparts is thus necessary to further elaborate how this fungus have adapted to its lifestyles and ecological niches in *E. chinensis*.

The three *Penicillium* species examined in this study show similar metabolic and enzymatic potential, alternatively, *Penicillium* spp. have also been reported to form fungus-farming mutualism with the other two *Euops* leaf-rolling weevils, *Euops lespedezae* (Kobayashi et al., 2008) and *Euops splendida* (Sakurai, 1985). *Penicillium* are one of the most chemically inventive genera, and are well known for their ability to produce a wide range of secondary metabolites and small molecules that function as antibiotics, toxins and pigments (Nielsen et al., 2017). The most well-known member of the genu is *Penicillium chrysogenum* because of its role in the production of penicillin and as a contaminant of indoor environments, food, and feedstuffs (van den Berg et al., 2008). *Penicillium decumbens* has been widely used in biorefinery due to its high production of cellulase and hemicellulose (Liu et al., 2013). The large enzymatic and secondary metabolic potential of *Penicillium* fungi (Nielsen et al., 2017) is consistent with their wide existence in natural ecosystems (Yadav et al., 2018), and also makes them an ideal candidate cultivar that can be recruited by fungus-farming insects.

The mutualistic *Euops*-*Penicillium* system shares a similar feature with those famous fungus-farming insects that they all depend fungi to produce the majority of the plant biomass-degrading enzymes. Genomic analyses of *Leucoagaricus gongylophorus*, a basidiomycetous fungus that serve as a food source for fungus-farming ants, have confirmed the presence of genes predicted to encode biomass-degrading enzymes for the digestion of cellulose, xylan and other plant polymers (Schiøtt et al., 2008), extracellular cellulases of *L. gongylophorus* include GH6 and GH7, GH15, and CE5 (Aylward et al., 2013). The fungus-farming termites primarily provision their fungal cultivars with decaying plant material, *Termitomyces* fungi and garden bacteria are responsible for lignin, cellulose, and hemicellulose degradation (Hyodo et al., 2003). The ambrosia fungi appear to preferentially degrade hemicellulose and other simple sugars (De Fine Licht and Biedermann, 2012). Using microbial symbiotes to access plant polysaccharides appears to be also shared by some nonsocial fungus-farming insects, such as a Eurasian woodwasp *Sirex noctilio* (F.) (Fu et al., 2020).

Transporter molecules are integral membrane proteins that facilitate movement of macromolecules, ions, or small molecules across a biological membrane (Perlin et al., 2014). MFS and ABC transporters are the two biggest families of fungal transporters that mediate transport of intermediates and toxic molecules in the secondary metabolism pathway (Perlin et al., 2014). In our study, although only a few ABC and MFS proteins from the three fungal species have been functionally characterized, we found that *P. herquei* had more ABC and MFS proteins than did the other two *Penicillium* species. Conversely, Fu et al. (2020) found that *Amylostereum areolatum* (Fr.) Boidin, a fungal symbiote of *S. noctilio*, had few ABC proteins in comparison with free-living fungi, possibly because *A. areolatum* is mainly transferred by its insect hosts. In contrast, although *P. herquei* is also carried in a special mycetangium by the female *E. chinensis* weevils before being used to inoculate leaf-rolls, it is also free-living after being inoculated on the leaf-rolls (Wang et al., 2015). Moreover, the leaf-roll is cut from the plant by the female weevil and drops to the soil, where it gradually decays in the moist environment (Li et al., 2012). Thus, *P. herquei* on the leaf-roll has a relatively long saprophytic phase in its life cycle, and thus it has evolved a strong ability to transport and discharge the intermediate and toxic substrates of leaf-rolls.

Maintaining monocultural fungus cultivar without other fungi present requires highly effective defense functions in fungus-farming systems (Schmidt et al., 2022). The production of toxic secondary metabolites by fungal symbiotes can provide protection to their hosts (Clay, 2014). *Penicillium herquei* can produce a vast array of biologically active secondary metabolites, including antibiotic phenalenones, norherqueinone and herqueinone, alkaloids, and some triene derivatives (Tansakul et al., 2014), that have shown significant antifungal and antiinfluenza activities (Zhou et al., 2019). We have demonstrated that *P. herquei* can suppress the growth of two pathogenic *Rhizopus* spp. frequently isolated from the leaf-rolls (Li et al., 2012). Wang et al. (2015) also found that *P. herquei* produces the antibiotic (+)-scleroderolide, which can protect the leaf-roll against potential infection. In line with this, our study found the *P. herquei* genome includes genes producing PKS, NRPS, T1PKS compounds, as well as terpenoid biosynthetic genes, which likely function in secondary metabolism and may serve to help the host to defend against toxins and parasites. PKS and NRPS are known to be symbiote-produced compounds that function in the defense of some insects and marine invertebrates (Piel, 2002). *Penicillium herquei* possesses a larger number of backbone genes associated with secondary metabolites, which may have antagonistic effects on pathogens and parasites that occur in both the leaf-rolls and the host insect. Notably, we found that *P. herquei* has significantly more genes for terpenoid biosynthesis than do the other two *Penicillium* species we studied. Terpenoids are the largest, most diverse group of secondary metabolites, exceeding phenolics, flavonoids, and alkaloids (Boncan et al., 2020). Terpenoids mediate various antagonistic and beneficial interactions among organisms (Gershenzon and Dudareva, 2007), and are used by many species of plants, animals and microorganisms to defend themselves against predators, pathogens, or competitors (Boncan et al., 2020). The possession of more terpenoid biosynthetic genes by *P. herquei* may contribute to higher terpene accumulation in *P. herquei*, leading to improved protection of leaf-rolls and *E. chinensis* larvae.

Pathogen-insect communications are essential for stimulating insect immune responses and inducing expression of virulence factors by fungal pathogens (Noman et al., 2021). Many studies have demonstrated that genes mediating pathogen-plant communications in signaling pathways are critical for fungal pathogenicity (Shang et al., 2016). The reduction in PHI genes in the *P. herquei* genome suggests that selection to maintain virulence genes has been relaxed, likely due to its mutualistic association with *E. chinensis*, and the fungal spores are carried in a special mycetangium on the female weevils. The female weevils protect the fungus before using it to inoculate the larval leaf-rolls. Moreover, *P. herquei* acts as an important nutritional resource for the *E. chinensis* larvae (Wang et al., 2010; Li et al., 2012). In this study, we found only a few virulence-associated genes (associated with increased virulence and lethal genes) in the *P. herquei* genome, and the inactivation or reduction of the expression of these genes can reduce or eliminate pathogenicity of this fungus to both the adults and the larvae of *E. chinensis*. Similarly, the absence of expression or low expression of lethal genes is also observed in the genome of the symbiote of the Eurasian wood wasp *S. noctilio* (Fu et al., 2020).

## Conclusion

The genome of *P. herquei* contains a diverse set of genes associating with carbohydrate-active enzymes, cellulose and hemicellulose degradation, transporter, and terpenoid biosynthesis. Comparative genomics demonstrate that the three *Penicillium* species show similar metabolic and enzymatic potential, however, *P. herquei* has more genes associated with plant biomass degradation and defense but less genes associating with virulence pathogenicity. Taken together, our results provide molecular evidence for plant substrate degradation and protective roles of *P. herquei* in its host *E. chinensis*. The large enzymatic and secondary metabolic potential shared by *Penicillium* genera is likely an important property that some *Penicillium* fungi are recruited by the *Euops* fungus-farming weevils as fungal crops.

## Data availability statement

The datasets presented in this study can be found in online repositories. The names of the repository/repositories and accession number(s) can be found at: https://www.ncbi.nlm.nih.gov/ GenBank: JANAWU000000000 BioProject: PRJNA854755 BioSample: SAMN29446570.

## Author contributions

XL and WG designed the experiments, analyzed the data, and wrote the paper. WW and WG sampled the leaf rolls. JT carried out fungal isolation. JT and TL contributed to the assembly of *P. herquei* and bioinformatics analysis. All authors contributed to the article and approved the submitted version.

## Funding

This work was supported by the National Natural Science Foundation of China (31800423 and 331460115) and the Natural Science Foundation of Guangxi Province (2018GXNSFBA281172).

## Conflict of interest

TL was employed by Wuhan Benagen Technology Company Limited.

The remaining authors declare that the research was conducted in the absence of any commercial or financial relationships that could be construed as a potential conflict of interest.

## Publisher’s note

All claims expressed in this article are solely those of the authors and do not necessarily represent those of their affiliated organizations, or those of the publisher, the editors and the reviewers. Any product that may be evaluated in this article, or claim that may be made by its manufacturer, is not guaranteed or endorsed by the publisher.

## Footnotes

^1^ https://github.com/BGI-flexlab/SOAPnuke

^2^ https://github.com/xiaochuanle/NECAT

^3^ http://www.repeatmasker.org

^4^ https://www.ncbi.nlm.nih.gov/refseq/about/nonredundantproteins/

^5^ http://darwin.uvigo.es/software/modeltest.html

^6^ http://tree.bio.ed.ac.uk/software/tracer/

^7^ http://www.phi-base.org/
