# Supplementary material for: Genome analysis and genomic comparison of a fungal cultivar of the nonsocial weevil Euops chinensis reveals its plant decomposition and protective roles in fungus-farming mutualism
Source: Front Microbiol. 2023 Feb 16;14:1048910. doi: 10.3389/fmicb.2023.1048910 (PMC9978505; doi:10.3389/fmicb.2023.1048910)
Supplement: Supplementary file 1 [file Data_Sheet_1.zip › Fig. S.docx]

**Fig. S1** GO functional annotation of coding sequences in the whole genome of *Penicillium herquei*.

**Fig. S2** The KEGG pathway annotation coding sequences in the whole genome of *Penicillium herquei.* The ordinate indicates the level-2 KEGG pathway classification, and the abscissa indicates the number of genes under the annotation of this classification. Different column colors represent the level-1 KEGG pathway classification. The rightmost bar indicates the number of genes under different level-1 classifications.

**Fig. S3** Protein orthologous comparison among genomes of *Penicillium herquei* and other 14 reference genomes.


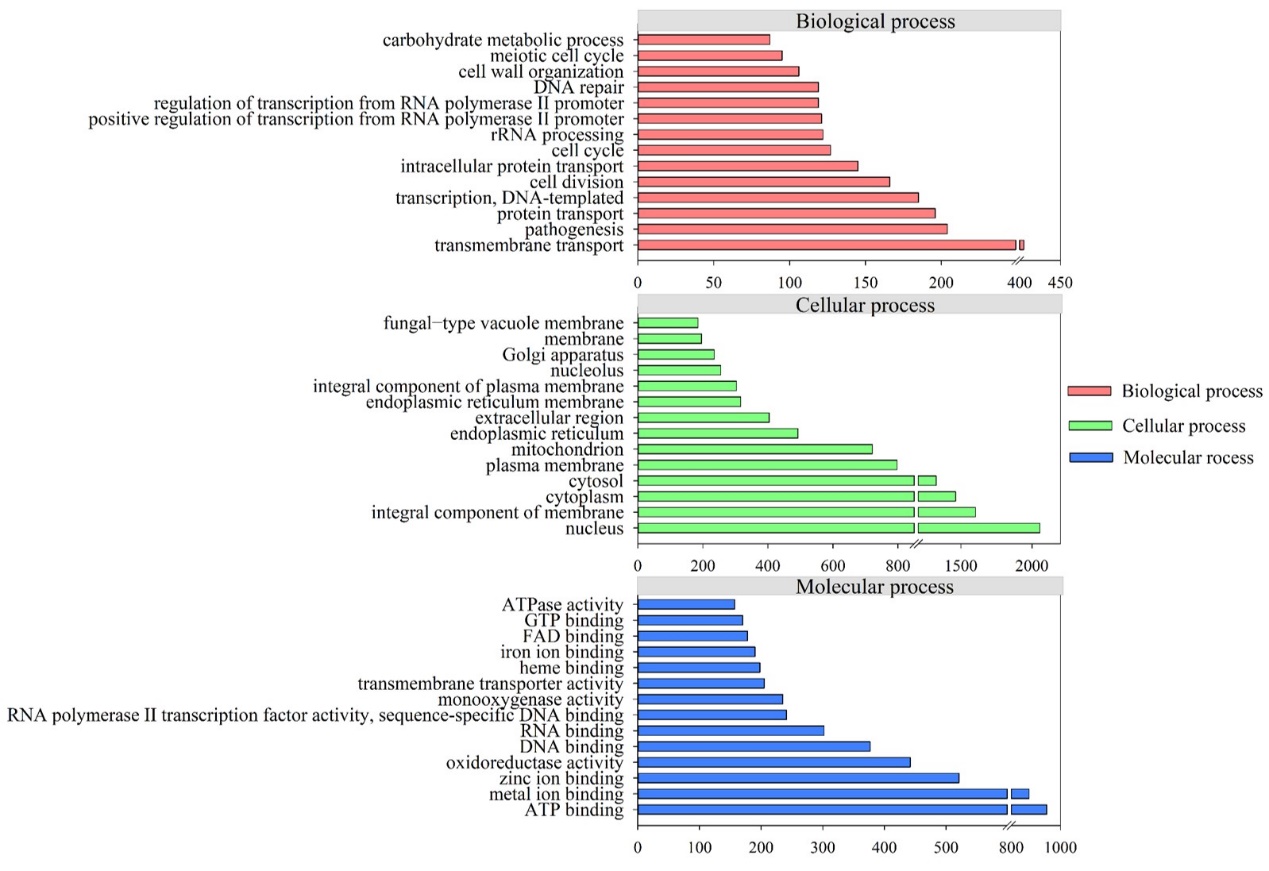


**Fig. S1**


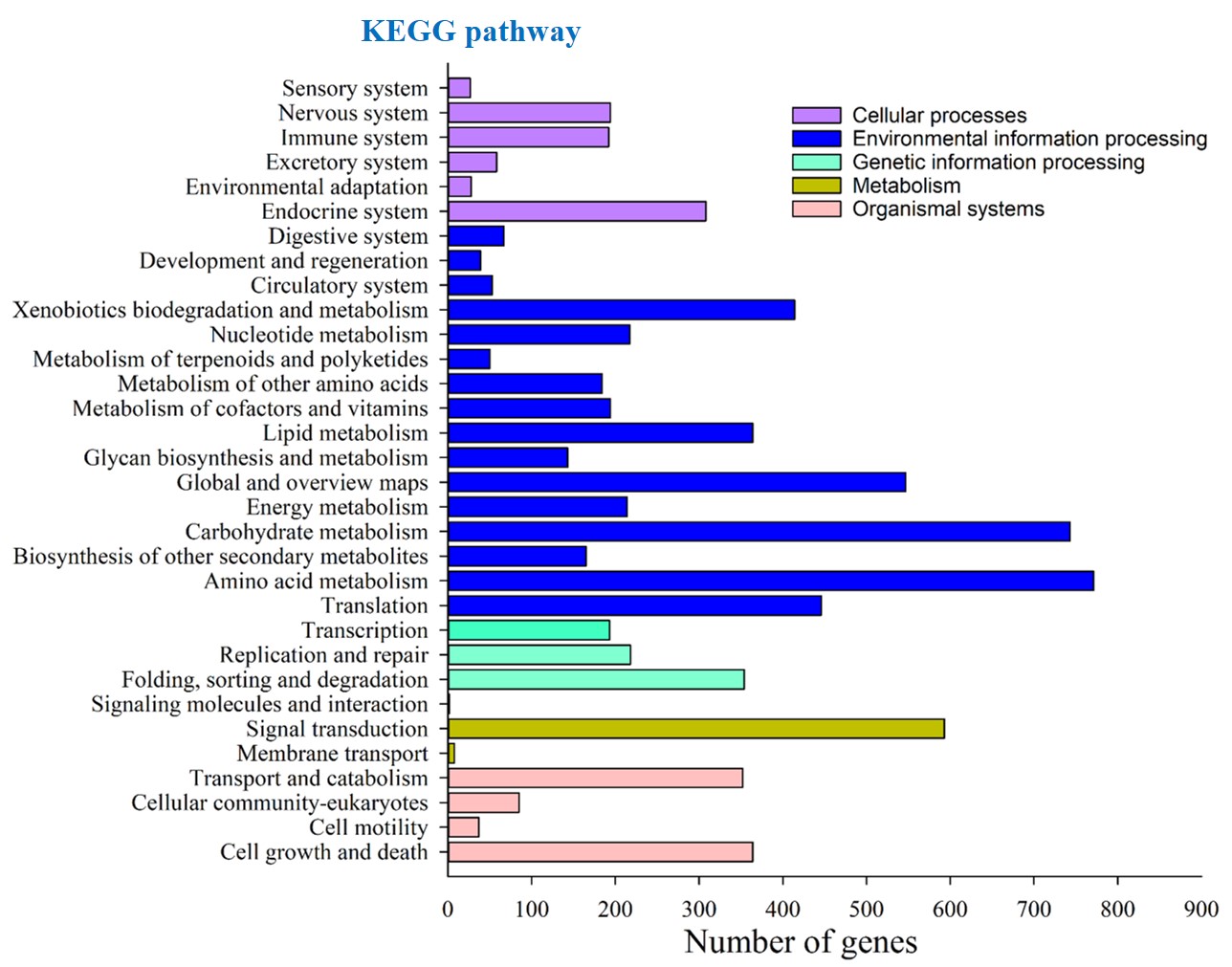


**Fig. S2**


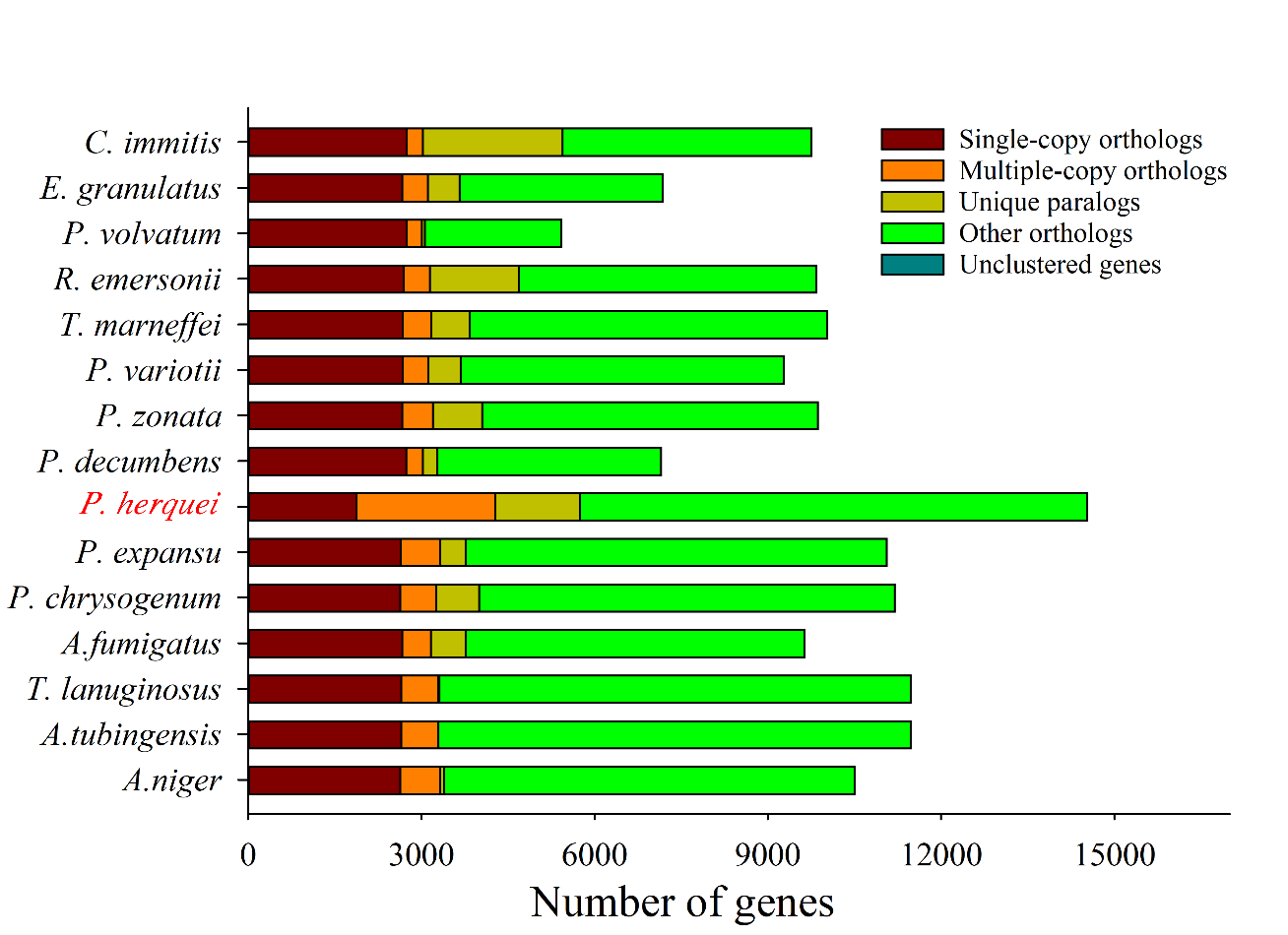


**Fig. S3**
