# Supplementary material for: Genome analysis and genomic comparison of a fungal cultivar of the nonsocial weevil Euops chinensis reveals its plant decomposition and protective roles in fungus-farming mutualism
Source: Front Microbiol. 2023 Feb 16;14:1048910. doi: 10.3389/fmicb.2023.1048910 (PMC9978505; doi:10.3389/fmicb.2023.1048910)
Supplement: Supplementary file 1 [file Data_Sheet_1.zip › Table S.docx]

**Table S1 Reference genomes for comparative analysis.**

| Species name | GenBank accession | Genome size (Mb) | #genes | %GC |
| --- | --- | --- | --- | --- |
| *Penicillium chrysogenum* | GCA_000149335.2 | 32.52 | 11198 | 48.9 |
| *Penicillium decumbens* | GCA_002072245.1 | 23.94 | 7149 | 50.2 |
| *Penicillium expansum* | GCA_000769745.1 | 32.36 | 11060 | 47.5 |
| *Aspergillus niger* | NC_007445.1 | 33.98 | 10785 | 50.4 |
| *Aspergillus fumigatus* | GCA_000002655.1 | 29.39 | 19260 | 49.8 |
| *Aspergillus tubingensis* | GCA_013340325.1 | 35.05 | 11898 | 49.3 |
| *Penicilliopsis zonata* | GCA_001890105.1 | 26.09 | 9870 | 49.9 |
| *Pseudotulostoma volvatum* | GCA_019804575.1 | 62.24 | 5417 | 45.8 |
| *Elaphomyces granulatus* | GCA_002240705.1 | 54.15 | 7171 | 45.6 |
| *Talaromyces marneffei* | GCA_000001985.1 | 28.64 | 10138 | 46.7 |
| *Thermomyces lanuginosus* | GCA_013340325.1 | 35.05 | 11809 | 49.3 |
| *Rasamsonia emersonii* | GCA_000968595.1 | 28.25 | 9843 | 50.6 |
| *Paecilomyces variotii* | GCA_004022145.1 | 30.11 | 9415 | 46.7 |
| *Coccidioides immitis* | GCA_000146045.2 | 29.02 | 9905 | 46.0 |

Note: all data are obtained from the National Center for Biotechnology Information (<https://www.ncbi.nlm.nih.gov/>) and the Fungal Genome Initiative website ([www.broad.mit.edu/annotation/fungi/fgi/](http://www.broad.mit.edu/annotation/fungi/fgi/)).

**Table S2 Statistic of gene families of 15 fungal genomes for comparative analysis.**

| Organism | Genes number | Genes in families | Family number | Unique families | Average genes per family |
| --- | --- | --- | --- | --- | --- |
| *P. herquei* | 14532 | 14532 | 9087 | 1077 | 1.6 |
| *P. chrysogenum* | 11198 | 11198 | 8856 | 436 | 1.26 |
| *P. decumbens* | 7149 | 7149 | 6490 | 243 | 1.1 |
| *P. expansum* | 11060 | 11060 | 8957 | 428 | 1.23 |
| *A. niger* | 10505 | 10505 | 8525 | 62 | 1.23 |
| *A. fumigatus* | 9630 | 9630 | 8369 | 579 | 1.15 |
| *A. tubingensis* | 11476 | 11476 | 9513 | 1 | 1.21 |
| *P. zonata* | 9869 | 9869 | 8397 | 818 | 1.18 |
| *P. volvatum* | 5417 | 5417 | 4969 | 40 | 1.09 |
| *E. granulatus* | 7171 | 7171 | 6064 | 363 | 1.18 |
| *T. marneffei* | 10023 | 10023 | 8234 | 543 | 1.22 |
| *T. lanuginosus* | 11476 | 11476 | 9513 | 2 | 1.21 |
| *R. emersonii* | 9843 | 9843 | 8541 | 1465 | 1.15 |
| *P. variotii* | 9270 | 9270 | 8094 | 537 | 1.15 |
| *C. immitis* | 9757 | 9757 | 8689 | 2072 | 1.12 |

**Table S3 Summary of annotation to each database.**

|  | Count | Percentage |
| --- | --- | --- |
| Annotation | 13,969 | 96.12% |
| Uniprot | 8,751 | 60.22% |
| Pfam | 11,765 | 80.96% |
| Refseq | 6,701 | 46.11% |
| Nr | 13,878 | 95.50% |
| Interproscan | 11,776 | 81.03% |
| GO | 8,665 | 59.63% |
| KEGG | 6638 | 45.67% |
| COG | 1,227 | 8.44% |

**Table S4 Transposable elements annotation.**

| Item | Number | Length (bp) | Coverage |
| --- | --- | --- | --- |
| SINE | 24 | 2,382 | 0.01% |
| LINE | 309 | 34,696 | 0.09% |
| LTR | 828 | 92,430 | 0.23% |
| DNA | 385 | 32,818 | 0.08% |
| Satellite | 43 | 4,438 | 0.01% |
| Simple repeat | 5,275 | 227,155 | 0.56% |
| Low complexity | 1,173 | 57,500 | 0.14% |
| Other | 86 | 8,487 | 0.02% |
| Unknown | 17 | 1,647 | 0.00% |
| Total | 8,140 | 458,687 | 1.14% |
